# Cross-Talk of NADPH Oxidases and Inflammation in Obesity

**DOI:** 10.3390/antiox12081589

**Published:** 2023-08-09

**Authors:** Henning Morawietz, Heike Brendel, Patrick Diaba-Nuhoho, Rusan Catar, Nikolaos Perakakis, Christian Wolfrum, Stefan R. Bornstein

**Affiliations:** 1 Division of Vascular Endothelium and Microcirculation, Department of Medicine III, University Hospital and Faculty of Medicine Carl Gustav Carus, TUD Dresden University of Technology, Fetscherstraße 74, 01307 Dresden, Germany; heike.brendel@ukdd.de (H.B.); diabanuh@uni-muenster.de (P.D.-N.); 2 Department of Paediatric and Adolescent Medicine, Paediatric Haematology and Oncology, University Hospital Münster, 48149 Münster, Germany; 3 Department of Nephrology and Critical Care Medicine, Charité-Universitätsmedizin Berlin, 10117 Berlin, Germany; rusan.catar@charite.de; 4 Department of Medicine III, University Hospital and Faculty of Medicine Carl Gustav Carus, TUD Dresden University of Technology, Fetscherstraße 74, 01307 Dresden, Germany; nikolaos.perakakis@ukdd.de (N.P.); stefan.bornstein@ukdd.de (S.R.B.); 5 Paul Langerhans Institute Dresden (PLID), Helmholtz Center Munich, University Hospital and Faculty of Medicine Carl Gustav Carus, TUD Dresden University of Technology, Fetscherstraße 74, 01307 Dresden, Germany; 6 German Center for Diabetes Research (DZD e.V.), Ingolstädter Landstrasse 1, 85764 Neuherberg, Germany; 7 Institute of Food, Nutrition, and Health, ETH Zürich, Schorenstrasse, 8603 Schwerzenbach, Switzerland; christian-wolfrum@ethz.ch; 8 Diabetes and Nutritional Sciences, King’s College London, Strand, London WC2R 2LS, UK

**Keywords:** antioxidants, cardiometabolic diseases, COVID-19, inflammation, obesity, non-alcoholic fatty liver disease, oxidative stress, reactive oxygen species

## Abstract

Obesity is a major risk factor for cardiovascular and metabolic diseases. Multiple experimental and clinical studies have shown increased oxidative stress and inflammation linked to obesity. NADPH oxidases are major sources of reactive oxygen species in the cardiovascular system and in metabolically active cells and organs. An impaired balance due to the increased formation of reactive oxygen species and a reduced antioxidative capacity contributes to the pathophysiology of cardiovascular and metabolic diseases and is linked to inflammation as a major pathomechanism in cardiometabolic diseases. Non-alcoholic fatty liver disease is particularly characterized by increased oxidative stress and inflammation. In recent years, COVID-19 infections have also increased oxidative stress and inflammation in infected cells and tissues. Increasing evidence supports the idea of an increased risk for severe clinical complications of cardiometabolic diseases after COVID-19. In this review, we discuss the role of oxidative stress and inflammation in experimental models and clinical studies of obesity, cardiovascular diseases, COVID-19 infections and potential therapeutic strategies.

## 1. Introduction

Cardiovascular and metabolic diseases are among the 10 most common causes of death globally [1]. A major risk factor of cardiometabolic diseases is obesity [2,3]. This is the result of a chronic imbalance of energy intake and energy expenditure resulting in excess fat storage [4]. Multiple experimental and clinical studies support the idea that increased levels of oxidative stress and inflammation are liked to obesity [5]. Oxidative stress is characterized by an impaired balance between increased formation of reactive oxygen species and reduced antioxidative capacity [6]. It contributes to the pathophysiology of cardiovascular and metabolic diseases [7,8] and is linked to inflammation as another major pathomechanism of cardiometabolic diseases [9,10]. Therefore, cardiovascular diseases and metabolic diseases such as type 2 diabetes or non-alcoholic fatty liver disease (NAFLD) can be described as inflammatory diseases with a chronic overload of free fatty acids and glucose that trigger inflammatory pathways via increased production of ROS [10,11].

NADPH oxidases are major sources of reactive oxygen species in the cardiovascular system and in metabolically active cells and organs [12,13,14,15,16]. Seven NADPH oxidase (Nox) isoforms have been described: Nox1–5 and dual oxidase (DUOX) 1–2 [11,17,18,19]. These are expressed in different cell types and tissues. The classical Nox isoform 2 (initially described as gp91phox) in phagocytes plays a particularly essential role in host defence against microbial pathogens and in the inflammatory reaction [20,21]. In the cardiovascular system, Nox1, Nox2 and Nox5 are considered as particularly major sources of free oxygen radicals such as superoxide anions [22,23]. Superoxide anions can inactivate nitric oxide and promote endothelial dysfunction and atherosclerosis [6,11,23]. On the other hand, physiological levels of hydrogen peroxide generated by Nox4 can mediate vaso- and cardioprotective mechanisms [24]. In this review, we will mainly discuss the impact of obesity on the different Nox isoforms and their implications for cardiometabolic diseases.

Non-alcoholic fatty liver disease (NAFLD) is an important risk factor of cardiovascular diseases [25]. NAFLD is characterized by increased oxidative stress and inflammation in the liver [26]. Increasing evidence supports the idea that there is an activation of Nox isoforms in models of NAFLD [27,28]. We will summarize these recent findings and discuss them as potential pathomechanisms of cardiometabolic diseases.

The COVID-19 pandemic has had a major impact on global health over the last 4 years [29]. Up to 50% of people who have died from COVID-19 had metabolic and vascular disorders [30]. COVID-19 infections accelerated the risk for severe clinical complications of cardiovascular and metabolic diseases [29,31]. Furthermore, COVID-19 infections increased oxidative stress and inflammation in infected cells and tissues [32]. We will discuss the role of oxidative stress and inflammation in experimental models and clinical studies of COVID-19 infections and its potential impact on cardiometabolic diseases.

**Figure 1:**
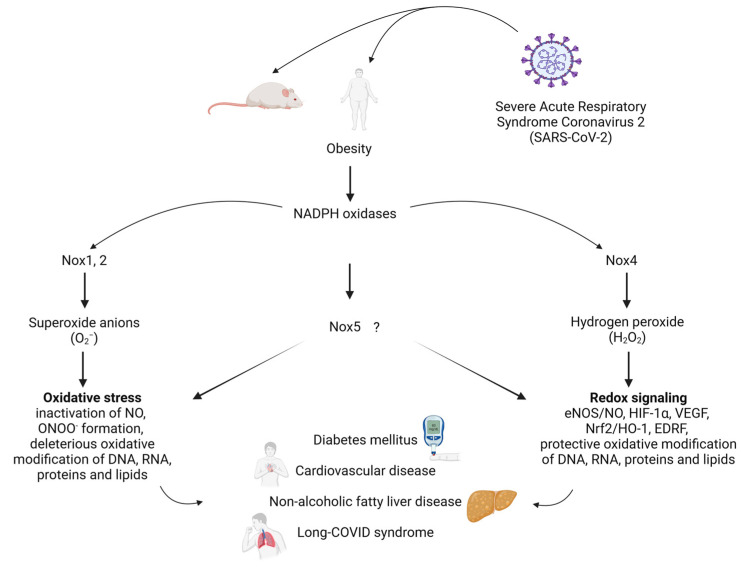
Impact of obesity and COVID-19 on NADPH oxidases (Nox). Nox isoforms as important sources of superoxide anions and hydrogen peroxide, oxidative stress and redox signalling in metabolic and cardiovascular diseases, non-alcoholic fatty liver disease and Long-COVID syndrome. Created with BioRender.com. Abbreviations: EDRF, endothelium-derived relaxing factor; eNOS, endothelial nitric oxide synthase; HIF-1α, hypoxia-inducible factor-1α; HO-1, heme oxygenase (decycling) 1; H_2_O_2_, hydrogen peroxide; NO, nitric oxide; Nox, NADPH oxidase; Nrf2, nuclear factor erythroid 2-related factor 2; O_2_^−^, superoxide anion; ONOO^−^, peroxynitrite; SARS-CoV-2, severe acute respiratory syndrome coronavirus 2; VEGF, vascular endothelial growth factor.

In summary, we will focus in this review on the role of NADPH oxidases as important sources of superoxide anions and hydrogen peroxide, oxidative stress and inflammation in experimental models and clinical studies of obesity, cardiovascular diseases, NAFLD and COVID-19 (Figure 1). Open questions and potential therapeutic strategies will be discussed.

## 2. NADPH Oxidases and Obesity

Reactive oxygen species (ROS) generated from NADPH oxidases have been associated with obesity and metabolic syndrome [33,34]. In an animal model, the membrane-bound subunit of NADPH oxidases p22phox was found to be essential for vascular ROS production and led to increased obesity after a high-fat diet [35].

However, the role of NADPH oxidases in obesity highly differs between various Nox isoforms and their intracellular and tissue location. Therefore, we summarize the knowledge about the role of different NOX isoforms in obesity and metabolic diseases in the following chapter.

### 2.1. NOX1

The role of NOX1 in obesity and metabolic diseases depends on the differential expression and localization of NOX1 in different cell types such as endothelial cells, smooth muscle cells and adipocytes.

Global Nox1^−/−^ mice were protected from cardiac hypertrophy after a high-fat and high-sucrose diet. Furthermore, Nox1 mediated endothelial activation and inflammation [36]. In *db/db* mice, deletion of Nox1 had no impact on body weight, fat mass and insulin resistance. However, endothelium-dependent vasorelaxation was improved in *db/db* mice lacking Nox1 [37]. The important role of Nox1 in the vasculature was further confirmed in apolipoprotein E deficient mice. Nox1 knockout did not alter body weight after atherogenic diet, but decreased superoxide anion burden and lesion formation in the aortic arch of these mice [38]. An association between NOX1, superoxide anion formation and obesity has also been observed in other tissues. Overproduction of ROS from NOX1, but not NOX2 or NOX4, has been reported in abdominal adipose stem cells [39]. In rats fed a high-fat diet, increased superoxide anion generation in the renal cortex was accompanied by an upregulation of Nox1, while Nox4 was downregulated. This was associated with elevated expression of inflammatory signalling molecules tumour necrosis factor-alpha (TNFα), cyclooxygenase-2 (COX2) and monocyte chemoattractant protein-1 (MCP-1) [40].

### 2.2. NOX2

The first evidence had been presented as early as 2009 that obese patients have higher gp91^phox^ (NOX2) expression and that patients with a hereditary deficiency of gp91^phox^ revealed higher flow-mediated arterial dilation [41]. In a clinical study with 100 participants, patients with obesity and hypercholesterolemia showed a significantly higher NOX2 expression compared with healthy subjects [42]. In obese Zucker rats, endothelial dysfunction was observed and associated with elevated TNFα expression and Nox2 activity in the perivascular adipose tissue [43]. In human adipose microvascular endothelial cells, insulin treatment increased NOX2 expression and superoxide anion generation. Additionally, the group detected impaired vascular function after insulin treatment ex vivo, which was reversible by NOX2 inhibition [44].

In several animal studies, deletion of Nox2 diminished or even abrogated obesity-induced deleterious effects. In global Nox2^−/−^ mice fed a high-fat diet for 16 weeks, manifestation of obesity, insulin resistance, dyslipidemia and endothelial dysfunction was absent [45]. In a study from Rahman et al., deficiency of Nox2 reduced the high-fat-diet-induced number of adipocytes and inflammatory cytokines in the bone marrow [46]. Deletion or pharmacological inhibition of Nox2 also diminished high-fat-diet-induced ventricular dysfunction in a mouse model [47]. While these studies were performed in global Nox2^−/−^ mice, Pepping et al. also found a reduced body weight, visceral inflammation and adiposity in myeloid lineage cell-specific Nox2 knockout mice compared with control mice after 16 weeks of a high-fat diet [48].

### 2.3. NOX4

NOX4 can also be found in different cells and tissues, including endothelial cells and adipocytes. Insulin-induced differentiation of adipocytes is accompanied by increased NOX4 and NOX1 expression. However, only inhibition of NOX4 by siRNA reduced adipocyte differentiation and ROS generation [49].

Nutrient supply with glucose and palmitate led to increased NOX4 activation in adipocytes. Similarly, Den Hartigh et al. found an upregulation of Nox4 in epididymal adipocytes after 8 weeks of high-fat and high-sucrose diet. In this study, adipocyte-specific Nox4 deficient mice revealed delayed onset of diet-induced adipose tissue inflammation and insulin resistance [50]. The obesity-reducing effect of the treatment with dihydroartemisinin (a malaria medication) has also been attributed to decreased Nox4 expression and subsequent attenuated adipocyte differentiation and lipid accumulation [51].

Li et al. have described how the deletion of Nox4 led to elevated adipose tissue accumulation and inflammation. Deletion of Nox4 further facilitated the high-fat-diet-induced insulin resistance in adipose tissues. Moreover, they reported diet-induced steatosis in the liver of Nox4^−/−^ mice [52].

Due to its predominant expression in endothelial cells, the role of NOX4 has been studied in more detail in the vasculature of models of obesity [53,54,55]. Schürmann et al. crossed Nox4 knockout mice with ApoE^−/−^ mice and found no differences between ApoE^−/−^ and double knockout mice in body weight, triglyceride or cholesterol concentrations after high-fat diet. However, depletion of Nox4 caused increased atherosclerosis in the aortic sinus and common carotid artery [56]. In parallel experiments, our group crossed Nox4 knockout mice with atherosclerosis-prone Ldlr^−/−^ mice to study the impact of Nox4 on atherosclerosis development in this genetic background. The weight gain during 20 weeks of high-fat diet did not differ between mice with or without Nox4. The observed endothelial dysfunction and resulting atherosclerosis, however, was more profound in mice lacking Nox4 [57]. Furthermore, in partial contrast with Li et al., we did not observe differences in diet-induced obesity between wild-type and Nox4^−/−^ mice. However, in a follow-up study, we found an essential role of Nox4 mediating the beneficial effects of exercise in the vasculature and skeletal muscle in obese mice fed a high-fat diet [58].

### 2.4. NOX5

NOX5 is a major NADPH oxidase isoform releasing ROS in human vessels [59]. In patients with coronary artery disease, NOX5 expression and activity was found to be elevated in coronary arteries [60]. The role of NOX5 in obesity is, so far, not well characterized, particularly because the NOX5 gene is absent in rodents. However, studies in humanized NOX5 knock-in mice have given novel insights into the possible role of NOX5 in obesity.

Humanized NOX5 knock-in mice showed reduced body weight gain and adipose mass after high-fat diet. In addition, applying conditional media from NOX5-expressing endothelial cells on 3T3-L1 adipocytes led to reduced lipid accumulation and increased glucose uptake [61]. The same group reported that NOX5 expression in obesity led to elevated interleukin (Il-6) production, which results in upregulated thermogenesis and lipolysis in adipose tissue via upregulation of Pgc1α and Ucp1 expression [62].

In summary, the Nox isoforms show specific roles in experimental models and human subjects with obesity and cardiometabolic diseases (Table 1). While Nox1 and Nox2 mainly promote deleterious effects in adipocytes and the vasculature, Nox4 seems to be a vasoprotective Nox isoform. The role of Nox5 is still under debate. While the elevated vascular NOX5 expression and activity in patients with coronary artery disease support the idea of a deleterious role in cardiovascular diseases, the reduced body weight gain and adipose tissue mass in humanized NOX5 knock-in mice suggest an additional protective role in obesity.

## 3. Cross-Talk of NADPH Oxidases, Inflammation, Hypercholesterolemia and Obesity

Multiple lines of evidence support the idea of a cross-talk between NADPH oxidases, inflammation, hypercholesterolemia and obesity [63]. This cross-talk might be specific for the different NOX isoforms. NADPH oxidases Nox1, Nox2 and Nox5 mainly generate superoxide anions, while Nox4 directly produces hydrogen peroxide [16]. Superoxide anions can interact with nitric oxide (NO), thus reducing the cardioprotective NO availability and forming cytotoxic peroxynitrite. They can also oxidatively modify a variety of biomolecules such as DNA, RNA, proteins and lipids [6]. H_2_O_2_ is considered an important signalling molecule due to its comparably long half-life and its ability to pass through membranes. The responses to ROS are dose dependent. Higher or lower ROS can have different, sometimes even opposite effects. Lower H_2_O_2_ concentrations (in the nanomolar range) are cardioprotective, while higher H_2_O_2_ concentrations (usually above 100 µm) can be cytotoxic and severely affect cell viability. In this context, the antioxidative capacity and the expression of antioxidative enzymes such as the three superoxide dismutase isoforms also have to be considered in specific cells and tissues [64]. The expression and activity of different Nox isoforms can be regulated on the transcriptional, translational and posttranslational level. Specific transcription factors can modulate the expression of Nox1, Nox2 and Nox4. Suppression of nuclear factor-kappaB (NF-κB) and the stimulation of inhibitor of kappaB (IκB) by troglitazone mediate an anti-inflammatory and potentially antiatherosclerotic effect in obese subjects [65]. On the posttranscriptional level, phosphorylation or dephosphorylation of different Nox isoforms have been shown to regulate the assembly and activity of Nox complexes [66]. Recently, the regulatory protein Poldip2 was found to act as an isoform-specific tunable switch for the activities of different NADPH oxidases. This selective regulatory role of Poldip2, positive for Nox4 or negative for Nox2, could regulate the type and level of ROS generated by p22^phox^-containing Nox isoforms in cells and tissues [67].

Strong evidence links Nox-generated ROS with vascular inflammation [68]. Stimulation of macrophages with cytokines such as IL-1, IL-6 or TNF-α induces the Nox2 complex and superoxide anion formation [11]. The results of multiple in vitro and in vivo studies support these findings. Recently, the chemokine C-C motif ligand 8 has been shown to promote atherosclerosis via Nox2-generated ROS inducing endothelial permeability [69]. The cytosolic subunit NADPH oxidase organizer 1 (NoxO1) of the Nox1 complex has also been linked to inflammation and atherosclerosis. These effects are gender specific. A reduced pro-inflammatory cytokine signature was found in the plasma of female but not male NoxO1^−/−^ mice. Furthermore, deletion of NoxO1 reduced atherosclerosis formation in brachiocephalic artery and aortic arch in female but not male NoxO1^−/−^ mice as compared with WT controls [70]. Systemic markers of oxidative stress and inflammation were elevated in insulin resistant and diabetic rats [71]. Roux-en-Y gastric bypass decreased hepatic NOX2, PKC-ζ, TNF-α expression and activation of NF-κB. Free fatty acids increased ROS formation, TNF-α and NOX2 protein expression, and activated NF-κB. Rosiglitazone attenuated the free-fatty-acid-induced increase in reactive oxygen species, TNF-α, NOX2, and NF-κB [72]. Antioxidants from raspberries and blackberries act in a synergistic manner to improve cardiac redox proteins (Nox1, Nox2) and reduce NF-κB and SAPK/JNK in mice fed a high-fat, high-sucrose diet [73].

We have been studying the cross-talk between NADPH oxidases, hypercholesterolemia and obesity for many years. A special focus of our research is the lectin-like oxidized low-density lipoprotein (oxLDL) receptor-1 (LOX-1) [74]. LOX-1 was initially described in endothelial cells [75], but can also be found in, e.g., vascular smooth muscle cells and macrophages. There is also a soluble form of LOX-1 (sLOX-1) which might be a molecular marker of cardiometabolic diseases. In previous studies, we have found inductions of LOX-1 by angiotensin II [76] and endothelin-1 [77]. Recently, we obtained a more detailed insight into the molecular mechanisms of the induction of LOX-1 by oxLDL [78]. These activation processes involved phosphorylation of protein kinases ERK1/2 and p38 MAPK, followed by activation of the transcription factor AP-1 and its binding to the promoters of the respective LOX-1 receptor gene. OxLDL-induced LOX-1 mRNA expression was abolished by a blockade of ERK1/2, p38 MAPK or AP-1. Furthermore, oxLDL, but not native LDL, was found to induce LOX-1 through an NF-κB-dependent pathway. These observations indicate that, in arterial endothelial cells, oxLDL signals primarily via LOX-1 receptors, which may accelerate endothelial dysfunction and atherosclerosis [78].

More recently, we were able to analyse the cross-talk between obesity, angiotensin II (Ang II), NOX2 and endothelin-1 (ET-1) in more detail [79]. Feeding mice a high-fat diet increased cardiac expression and plasma levels of Ang II and ET-1 in wild-type but not in Nox2-deficient animals. Exposure of human endothelial cells to Ang II resulted in increased ET-1 production, which could be blocked by silencing human NOX2. Ang II induced NOX2 expression, through induction of the Oct-1 (human/mouse octamer binding transcription factor 1 protein) and activation of the NOX2 promoter region containing Oct-1-binding sites. Induction of NOX2 expression by Ang II was associated with increased production of superoxide anions. Inhibition of Oct-1 by small interfering RNA reduced Ang II-induced NOX2 expression and superoxide anion production, and neutralization of superoxide by superoxide dismutase abolished Ang II-stimulated human ET1 promoter activity, pre-pro ET1 mRNA expression, and ET-1 release. In summary, Ang II may promote ET-1 production in the endothelium of obese mice in response to atherogenic diets through a mechanism that involves the transcription factor Oct-1 and the increased formation of superoxide anions by NOX2 [79].

In conclusion, our data support the idea of a vicious cycle of increased NADPH oxidases and ROS formation, oxidative modification of LDL to oxLDL, increased uptake of oxLDL by LOX-1 and subsequent induction of NADPH oxidases, Ang II and ET-1 expression. Ang II and ET-1 further promote this pro-inflammatory and oxidative vicious cycle.

## 4. Oxidative Stress and NADPH Oxidases in Non-Alcoholic Fatty Liver Disease

Non-alcoholic fatty liver disease (NAFLD) affects more than 30% of the general population and is considered to be the hepatic component of the metabolic syndrome [80]. Excessive caloric intake leads to increased transportation both of dietary fat to the liver as well as of carbohydrates that will be converted to fat by de novo lipogenesis [26,81,82]. Moreover, increased adipose tissue lipolysis observed in obesity will also result in higher concentrations of circulating free fatty acids and increased uptake of them by the liver [26,81,82]. Liver fat will be initially stored in the form of triacylglycerols (TGs) which are the main component of intracellular lipid droplets. This condition has been named “steatosis” and liver fat accumulation serves as a protective mechanism against hepatocyte injury in the beginning. However, as liver fat uptake continues, the fat storage and fat removal capacities of the liver are exhausted, resulting in the accumulation of toxic lipid species (lipotoxicity), such as ceramides, saturated fatty acids and lysophosphatidylcholines [26,81,82,83,84,85]. These species induce ROS production by activating oxidative phosphorylation and β-oxidation in mitochondria, by impairing antioxidant response, as well as by inducing endoplasmatic reticulum stress. Increased ROS production will contribute to hepatocellular damage or death as well as to the release of damage-associated molecular patterns (DAMPs), cytokines, hormones and lipids [26,81,82,83,84,85]. The released factors will promote hepatic inflammation by recruiting and activating both peripheral and resident macrophages. This condition, named “non-alcoholic steatohepatitis” (NASH), is observed in approximately 20% of the patients with NAFLD. As hepatic inflammation further progresses, hepatic stellate cells are activated, leading to fibrogenesis and consequently to liver fibrosis, and, in more advanced stages, to liver cirrhosis as well as to hepatocellular carcinoma (HCC) [26,81,82,83,84].

Given the crucial role of NADPH oxidases (NOXs) in ROS production, several studies have evaluated to what extent NOXs participate in the progression of steatosis to NASH or to liver fibrosis and HCC. NOX1 expression has been shown to increase in patients with NASH as well as in mice with NASH due to a high fructose and high cholesterol diet [86]. This upregulation in NOX1 is observed primarily in liver sinusoidal endothelial cells (LSECs) when they are exposed to saturated fatty acids and leads to increased production of ROS. The NOX1-induced ROS seems to directly cause hepatocellular injury as well as to indirectly cause it through the reduction of NO bioactivity and thus impaired microcirculation [86]. The DAMPs and cytokines released from the injured hepatocytes may lead to an increase in the expression of NOX1 by macrophages that will enhance the production of inflammatory cytokines and facilitate liver tumorigenesis [27,28,86,87]. Finally, ROS deriving from NOX1 may stimulate the proliferation of hepatic stellate cells, thus promoting the development of liver fibrosis [88].

Similar effects have been also reported for NOX2. NOX2 can induce premature senescence of LSECs, promoting fibrogenesis [89]. Furthermore, saturated fatty acids induce NOX2-dependent ROS production in peripheral macrophages migrating to the liver (but not in resident macrophages, i.e., Kupffer cells) which may promote hepatic inflammation, steatosis and insulin resistance [90]. NOX2 also seems to trigger the polarization of tumour-associated macrophages (TAMs) to an M2 phenotype that further induces tumour growth in HCC [91]. Apart from these direct effects on the liver, NOX2 may affect liver function indirectly. Specifically, NOX2 seems to modulate the inflammatory properties and adipocyte-clearing potential of macrophages and may thus regulate adipose tissue function [92,93]. NOX2-driven changes in adipose tissue may impair insulin sensitivity, glucose and lipid homeostasis and thus indirectly affect liver fat accumulation and liver inflammation [92,93].

NOX4 has also been implicated in NAFLD pathophysiology. NOX4 is predominantly expressed in hepatocytes and its expression is increased in patients with NASH and in mice that develop steatosis and fibrosis due to a fast-food diet or to a choline-deficient diet [94]. Hepatocyte-specific knockout of NOX4 or administration of a NOX4 inhibitor in mice reduces liver inflammation and fibroses and improves insulin sensitivity [94]. NOX4 expression is also increased in HCC cells and expression levels correlate with short overall survival [95]. NOX4 has been associated with alterations in cell proliferation and apoptosis through regulation of the TGF-β activity [96,97]. Dual inhibition of NOX1 and NOX4 with setanaxib (GKT-831) has been shown to result in increased cell cytotoxicity by induction of apoptosis in HCC cell lines, thus identifying a compound with antitumorigenic properties which deserves further evaluation [98].

NAFLD remains an unmet clinical need, with no approved treatment to date. NOXs are involved in different stages of NAFLD, from inflammation to fibrosis and to HCC development. Thus, they seem to be promising targets for the development of drugs that will be potentially capable of modulating hepatic inflammation and fibrogenesis and consequently improve or delay NAFLD progression.

## 5. COVID-19, Obesity, Inflammation and Oxidative Stress in Cardiometabolic Disorders

The COVID-19 pandemic has had a severe impact on cardiovascular and metabolic morbidity and mortality in the last years [31]. Severe acute respiratory syndrome coronavirus 2 (SARS-CoV-2) infections could lead to local and systemic inflammation resulting in a so-called “cytokine storm” with life-threatening complications [99]. Type 2 diabetes mellitus and hypertension have been the most common comorbidities in patients with coronavirus infections [100]. We were part of a team reporting the first manifestations of insulin-dependent diabetes to occur following SARS-CoV-2 infection in a young individual in the absence of the autoantibodies that are typical of type 1 diabetes mellitus [101]. New-onset diabetes and severe metabolic complications of preexisting diabetes, such as diabetic ketoacidosis and hyperosmolarity, have been observed in patients with COVID-19 [102]. We could detect SARS-CoV-2 viral infiltration of beta-cells in COVID-19 patients and were able to show that SARS-CoV-2 pseudoviruses can infect isolated human islet cells [103]. COVID-19 has short-term and long-term effects on patients with diabetes mellitus [104]. We could further show that the SARS-CoV-2 virus also targets human adrenal glands as major stress organs [105]. The adrenal gland could also play an important role in Long-COVID-19 syndrome [106].

Obesity is an independent risk factor of COVID-19 severity and mortality [107]. Several mechanisms could be responsible for the increased risk of severe COVID-19 and mortality in obese patients [108]. Patients with obesity have an increased risk of pulmonary fibrosis, chronic obstructive pulmonary disorder, and reduced respiratory function [109]. Many adipose patients have an impaired immune system. Obesity is characterized by hyperplasia and hypertrophy of adipocytes and accumulation of macrophages in the adipose tissue [110]. A switch from an anti-inflammatory M2 type to the pro-inflammatory M1 form of macrophages is observed in obesity [111]. This is supported by the increased expression of pro-inflammatory cytokines such as TNF-α, IL-6 and IL-1β in the adipose tissue of obese patients. Therefore, COVID-19 and obesity may lead to a state of chronic inflammation [112].

There are also links between COVID-19 and the lipid metabolism [113]. An elevated cholesterol concentration has been suspected to increase the susceptibility for SARS-CoV-2 infection. During COVID-19, LDL cholesterol and HDL cholesterol appear to be decreased. On the other hand, triglycerides are elevated. Extracorporeal apheresis has been suggested to alleviate symptoms of Post-COVID syndrome [114]. We have recently been able to show that therapeutic apheresis reduces the concentration of neurotransmitter autoantibodies, lipids, and inflammatory markers in patients with Long-COVID [115]. This could provide a novel therapeutic strategy in the treatment of patients with Long-COVID syndrome.

COVID-19 can also have deleterious effects on liver function. It may affect the development and progression of NAFLD, which can in turn further aggravate the consequences of a COVID-19 infection [116].

What are the potential implications of COVID-19 on NADPH oxidases and oxidative stress? A potential role of increased oxidative stress in cardiovascular injury and antioxidative treatment options for COVID-19 was proposed early in the pandemic [117]. Indeed, increased oxidative stress by Nox2 activation has been shown in COVID-19 patients and is associated with severe disease and thrombotic events [118]. NADPH oxidases might be interesting therapeutic targets in infectious and inflammatory diseases [119]. 17β-estradiol completely reversed S protein-induced activation of NOX2 and reactive oxygen species production, angiotensin-converting enzyme 2 (ACE2) upregulation and induction of pro-inflammatory monocyte chemoattractant protein-1 (MCP-1) in endothelial cells, thus attenuating endothelial dysfunction [120]. More recently, NOX2 and NOX5 have been shown to be increased in the cardiac microvascular endothelium of deceased COVID-19 patients, which may contribute to their previously reported cardio-microvascular dysfunction [121]. Recently, it has been shown that patients with COVID-19 have significant higher markers of oxidative stress (such as soluble Nox2-derived peptide and hydrogen peroxide) and inflammation (TNF-α and IL-6), while, conversely, flow-mediated dilation, hydrogen peroxide breakdown activity and nitric oxide (NO) bioavailability were shown to be significantly lower [122].

Several antioxidants and potentially antioxidative agents have been discussed as therapeutic strategies in the context of COVID-19 [123]. Vitamin D has been shown to induce the overexpression of glutathione, glutathione peroxidase, and superoxide dismutase, and the down-regulation of NADPH oxidase, to reduce oxidative stress and it may also be effective in the prevention of complications of COVID-19 [124]. This concept has been controversially discussed [125]. Polyphenols, which have been proposed to serve in the prevention and therapy of COVID-19 due to their anti-inflammatory properties, also have an inhibitory effect on NOXs activity [126]. Antioxidants and plant ingredients such as quercetin, glabridin, gallic acid and chrysoeriol could inhibit SARS-CoV-2 spike protein (S protein)-induced upregulation of its target molecule ACE2 in endothelial cells, S protein-induced upregulation of NOX2 protein expression and superoxide production and of pro-inflammatory MCP-1 expression [127]. Therefore, these small molecules may be used as novel therapeutic options for the treatment of patients with COVID-19 by prevention of the S protein induction of endothelial oxidative stress and inflammation. Furthermore, antioxidants may prevent renal-damage-induced cell death by inhibiting oxidative damage and COVID-19 related comorbidities [128].

(−)-Epigallocatechin-3-gallate is a major polyphenol of green tea and acts as a strong antioxidant which scavenges reactive oxygen species, inhibits pro-oxidant enzymes including NADPH oxidase, and activates antioxidant systems including superoxide dismutase, catalase, or glutathione. It also mediates potent anti-inflammatory and metabolic effects and might be beneficial in respiratory diseases with acute or chronic inflammatory and oxidative processes, such as COVID-19 [129].

## 6. Clinical Implications

Several novel therapeutic options for type 2 diabetes have been developed in recent years, and they should ideally target hyperglycemia, insulin resistance and obesity [130]. Excellent therapeutic options are Glucagon-like peptide-1 receptor agonists (GLP-1 RAs) and the new GLP1 and gastric inhibitory polypeptide dual receptor antagonists counteract metabolic defects of type 2 diabetes, hyperglycemia and obesity. The results are very promising and similar to the effects of bariatric surgery [131]. The new “magic bullet” in the treatment of metabolic, renal and cardiovascular diseases are the sodium-glucose cotransporter 2 (SGLT2) inhibitors [132]. These improve the clinical symptoms and outcome of patients with diabetes, diabetic nephropathy and cardiovascular diseases such as heart failure with reduced ejection fraction [133]. SGLT2 inhibitors were even recently approved as the first form of medication for the rapidly growing number of patients with heart failure and preserved ejection fraction [134]. GLP-1 receptor antagonists and novel drugs currently in development might be even more promising because they combine antihyperglycemic effects with remarkable weight loss [135,136].

The COVID-19 pandemic has been a severe threat to the health of millions of patients worldwide. Because patients with diabetes have an increased risk of severe complications of SARS-CoV-2 infections, including adult respiratory distress syndrome and multi-organ failure, we have formed an international panel of experts in the field of diabetes and endocrinology to provide some guidance and practical recommendations for the management of diabetes during the pandemic [137]. In 2021, we updated these practical recommendations for the management of patients with COVID-19 and post-pandemic clinical complications [30]. For patients with obesity, diabetes and associated cardiovascular diseases, the closure and slow reuptake of bariatric (metabolic) surgery posed an inappropriate risk. In most countries, elective surgery procedures have been suspended to preserve hospital resources for COVID-19 treatment. Therefore, the waiting lists for bariatric surgery have been rapidly increasing [138]. Even while the vaccinations were very successful in reducing the number of acute SARS-CoV-2 infections, millions of patients still suffer from “post-COVID syndrome” or “long-COVID”. These patients have symptoms of chronic fatigue syndrome linked to a viral and autoimmune pathogenesis. In these disorders, neurotransmitter receptor antibodies against ß-adrenergic and muscarinic receptors may play a key role. An elevation of antibodies against ß-adrenergic and muscarinic receptor autoantibodies in these patients has been observed. Extracorporeal apheresis using a specific filter seems to be effective in reducing these antibodies and improving the symptoms of patients with chronic fatigue syndrome. Therefore, such neuropheresis may provide a promising novel therapeutic option for patients with post-COVID-19 syndrome [139].

**Figure 2:**
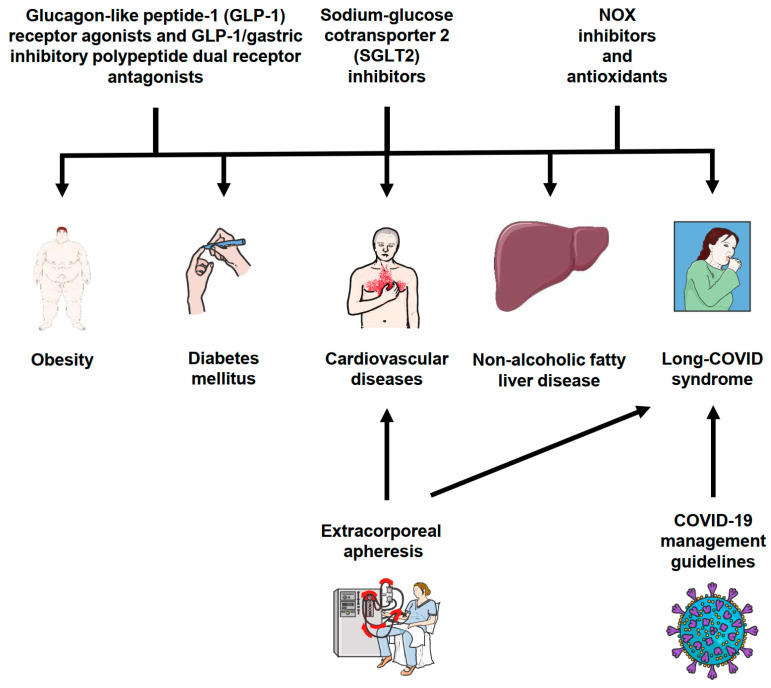
Schematic overview of potential therapeutic strategies for cardiometabolic complications of obesity and COVID-19. Parts of the figure are adapted from SMART—Servier Medical Art, Servier: https://smart.servier.com, accessed on 30 June 2023. Abbreviations: COVID-19, coronavirus disease 2019; GLP-1, glucagon-like peptide-1; NOX, NADPH oxidase; SGLT2, sodium-glucose cotransporter 2.

The potential therapeutic strategies for cardiometabolic complications of obesity and COVID-19 are summarized in Figure 2 and compared in Table 2.

## 7. Open Questions

In several animal models, antioxidant treatments have shown protective effects against oxidative stress, inflammation and deleterious metabolic and against the cardiovascular effects of obesity. On the other hand, the US Preventive Services Task Force does not support the use of single- or paired-nutrient supplements (other than beta carotene and vitamin E) for the prevention of cardiovascular disease or cancer [140]. The evidence for using antioxidants—such as in dietary berry intake to reduce inflammation and oxidative stress in cardiometabolic conditions—is still inconsistent in reported effectiveness and needs further investigation [141]. More selective isoform-specific inhibitors of NADPH oxidase may be potential therapeutic agents to improve the deleterious effects of obesity on cardiometabolic clinical parameters and function.

## 8. Conclusions

In conclusion, the results of a variety of experimental and clinical studies support the idea of an important causal role of NADPH oxidases, oxidative stress and inflammation in obesity, cardiovascular diseases, NAFLD and COVID-19. More selective antioxidants might be effective tools for decreasing specific Nox isoforms, ROS formation and inflammation, and might provide attractive therapeutic strategies in the prevention of obesity-associated cardiometabolic diseases.

## Figures and Tables

**Table 1:** Roles of different NOX isoforms in obesity and metabolic diseases.

NOX Isoform	Role in Obesity and Cardiometabolic Diseases	References
NOX1	Mediates endothelial activation, inflammation and cardiac hypertrophy; promotes microvascular dysfunction in *db/db* mice; increases superoxide anion formation in abdominal fat-derived mesenchymal stromal cells and renal cortex; acts as a link between obesity and kidney injury.	[36,37,38,39,40]
NOX2	Higher expression in obese patients; promotes endothelial dysfunction in obese rats; NOX2 deletion reduces obesity-induced effects; NOX2 inhibition improves vascular function.	[41,42,43,44,45,46,47,48]
NOX4	Anti-atherosclerotic, vaso- and cardioprotective role; involved in insulin-induced adipocyte differentiation; NOX4 inhibition reduces adipocyte differentiation and ROS generation; adipocyte-specific deletion delays diet-induced inflammation and insulin resistance; deletion causes adipose tissue accumulation and insulin resistance by high-fat diet.	[49,50,51,52,53,54,55,56,57,58]
NOX5	Elevated NOX5 expression and activity in coronary arteries of patients with coronary artery disease; limited knowledge about role in obesity, but reduced body weight gain and adipose tissue mass in humanized NOX5 knock-in mice suggest an additional protective role in obesity.	[59,60,61,62]

Abbreviations: *db/db* mice, mice homozygous for spontaneous mutation diabetes in leptin receptor (Lepr^db^); NOX, NADPH oxidase; ROS, reactive oxygen species.

**Table 2:** Potential therapeutic strategies for cardiometabolic complications of obesity and COVID-19.

Therapeutic Approach	Targeted Conditions/Effects	References
Glucagon-like peptide-1 receptor agonists (GLP-1 RAs) and GLP-1/gastric inhibitory polypeptide dual receptor antagonists	Hyperglycemia, insulin resistance, obesity	[130,131,135,136]
Sodium-glucose cotransporter 2 (SGLT2) inhibitors	Type 2 diabetes, hyperglycemia, diabetic nephropathy, cardiovascular diseases, heart failure with reduced ejection fraction	[130,132,133,134]
COVID-19 management guidelines	Guidance for diabetes management during the pandemic, post-pandemic complications, and risk reduction for SARS-CoV-2 infections	[30,137]
Extracorporeal apheresis	Potential therapeutic option for patients with COVID-19 and post-COVID-19 syndrome, targeting inflammatory and autoimmune pathogenesis by reducing proinflammatory mediators and ß-adrenergic and muscarinic receptor autoantibodies	[31,113,139]
NOX inhibitors and antioxidants	Selective inhibitors of NOX1 and 2 and antioxidants could reduce superoxide anion formation, oxidative stress, increase NO availability and reduce the deleterious oxidative modification of biomolecules	[6,16,17,22,28]

Abbreviations: COVID-19, coronavirus disease 2019; GLP-1 RAs, glucagon-like peptide-1 receptor agonists; NOX1, 2, NADPH oxidase 1, 2; NO, nitric oxide; SARS-CoV-2, severe acute respiratory syndrome coronavirus 2; SGLT2, sodium-glucose cotransporter 2.

## Data Availability

Not applicable.
